# Arabidopsis Transcription Regulatory Factor Domain/Domain Interaction Analysis Tool—Liquid/Liquid Phase Separation, Oligomerization, GO Analysis: A Toolkit for Interaction Data-Based Domain Analysis

**DOI:** 10.3390/genes14071476

**Published:** 2023-07-19

**Authors:** Jee Eun Kang, Ji Hae Jun, Jung Hyun Kwon, Ju-Hyun Lee, Kidong Hwang, Sungjong Kim, Namhee Jeong

**Affiliations:** Fruit Research Division, National Institute of Horticultural and Herbal Science, Wanju 55365, Republic of Korea; jekang_39@yahoo.com (J.E.K.);

**Keywords:** liquid–liquid phase separation, protein oligomerization, GO, domain–domain interaction, domain linker, intrinsically disordered regions, domain–peptide interaction, beta sheet, transmembrane helices, post-translational modification

## Abstract

Although a large number of databases are available for regulatory elements, a bottleneck has been created by the lack of bioinformatics tools to predict the interaction modes of regulatory elements. To reduce this gap, we developed the Arabidopsis Transcription Regulatory Factor Domain/Domain Interaction Analysis Tool–liquid/liquid phase separation (LLPS), oligomerization, GO analysis (ART FOUNDATION-LOG), a useful toolkit for protein–nucleic acid interaction (PNI) and protein–protein interaction (PPI) analysis based on domain–domain interactions (DDIs). LLPS, protein oligomerization, the structural properties of protein domains, and protein modifications are major components in the orchestration of the spatiotemporal dynamics of PPIs and PNIs. Our goal is to integrate PPI/PNI information into the development of a prediction model for identifying important genetic variants in peaches. Our program unified interdatabase relational keys based on protein domains to facilitate inference from the model species. A key advantage of this program lies in the integrated information of related features, such as protein oligomerization, LOG analysis, structural characterizations of domains (e.g., domain linkers, intrinsically disordered regions, DDIs, domain–motif (peptide) interactions, beta sheets, and transmembrane helices), and post-translational modification. We provided simple tests to demonstrate how to use this program, which can be applied to other eukaryotic organisms.

## 1. Introduction

Peaches (Prunus persica) have been bred for more than 4000 years [1]. Traditional breeding has facilitated the selection of peach cultivars with improved fruit quality and traits over thousands of years. Over the last several decades, marker-assisted breeding was developed based on advanced next-generation sequencing technologies and has gained popularity among breeding scientists [2]. Genome-wide association studies (GWASs) have been employed to improve marker-assisted breeding [2]. However, identifying important functional genetic variants in GWAS data remains challenging due to the high complexity of genetic variations. There are only limited resources available for peaches compared to the model species *Arabidopsis thaliana* (*A. thaliana*). Considerable portions of regulatory mechanisms have been conserved across plant species; for instance, the TF families of *A. thaliana* are subsets of those of peaches. To effectively solve this problem, we took a strategic approach: integration of the immense reservoir of omics data from the model species into the genetic variant analysis of peaches. Our long-term research plan has three stages. The first one is to automate gene regulatory network (GRN) construction with regulatory elements and interaction information. The second one is to integrate the GRN into an analysis of GWASs, RNA-seq, transcriptome-wide association studies (TWASs), epigenome-wide association studies (EWASs), and metabolic pathways from 1001 *A. thaliana* genome projects. The third one is to make inferences in selecting the phenotype-determinant genetic variants in the GWAS data from peaches. The content of this article is limited to introducing software developed for data-driven predictions of interaction modes of regulatory elements in the first stage.

Well-organized A. thaliana databases can expedite the process of GRN construction; regulatory elements of GRN nodes can be derived from various data, such as those found with 3did, Plant Regulomics, and iRegNet, in addition to the data of the genome-wide positions of domain and gene regions [3,4,5,6,7]. However, important pieces of information are missing: interactions of regulatory elements (e.g., domain structural elements in PPI or promoter motifs in PNI). This has caused a research bottleneck. For biological processes that employ complex regulatory systems, complete annotations of individual interactions are not available. However, integration of LOG analysis, domain–domain interactions (DDIs), domain–peptide interactions (DMIs), the physicochemical and structural properties of domains (e.g., transmembranes, domain linkers, intrinsically disordered regions (IDRs), DMIs, and coiled-coil regions), and post-translational modification (PTM) may have the power to predict and infer available PPI/PNI modes. For example, transcription regulation is triggered by cellular signals and achieved with the spatiotemporal coordination of various protein–nucleic acid interactions (PNIs) and protein–protein interactions (PPIs) that are regulated in a number of ways, e.g., protein oligomerization, liquid–liquid phase separation (LLPS), and PTM [8,9]. These factors have evolved into interwoven regulatory mechanisms that modulate interactions: for instance, repressing the PNIs of Histone H1 by inhibiting LLPS formation with phosphorylation, regulating binding activity between scaffold proteins and peptides with oligomerization and LLPS formation, and regulating the localization of self-assembled transmembrane proteins with phosphorylation of amino acids in domains [10,11,12,13,14,15,16,17,18,19,20,21,22].

**Figure 1:**
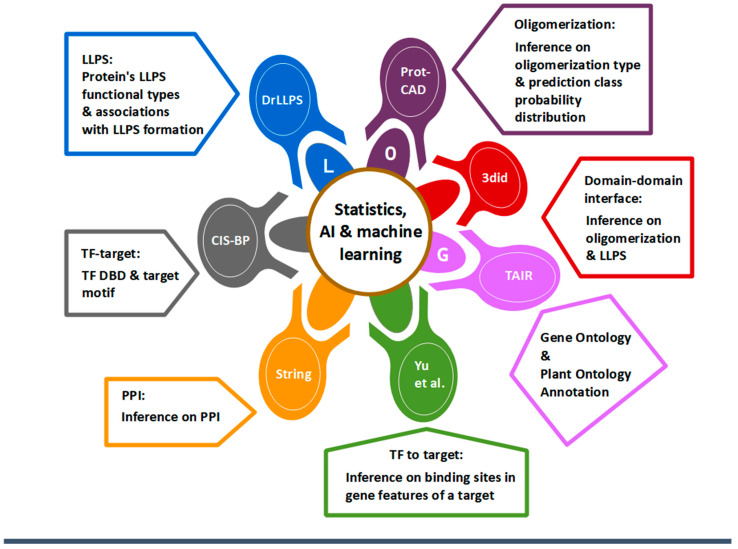
Main database modules in ART FOUNDATION-LOG [33].

Here, we introduced the Arabidopsis Transcription Regulatory Factor Domain/Domain Interaction Analysis Tool–LLPS, oligomerization, GO analysis (ART FOUNDATION-LOG): a useful toolkit with integrative resources on key properties that modulate PPIs and PNIs. This program consists of seven main modules, each of which was built based on existing databases: the protein oligomerization module (ProtCAD), the domain-binding interfaces of DDIs and domain characterization (3did, Plant-PrAS, and qPTMplants), LLPS formations (DrLLPS), GO/PO analysis, the TF binding profile module (Cis-BP), the PPI module (String), and the TF-target module (TF2DNA, Yu et al.) (Figure 1) [5,23,24,25,26,27,28,29,30,31,32,33,34,35,36,37,38]. 

This article mainly discusses the ART FOUNDATION-LOG and briefly demonstrates the integration of regulatory elements. To demonstrate how to use this program, we performed three simple tests. In the first and second tests, the average accuracies of the predictions of oligomerization and LLPS types were 75% and 76%, respectively. In the last test, we selected LLPS factors in signaling pathways and screened the TFs that were associated with those factors. The accuracy of predicting TF motif types was 91%. The integration of a regulatory element into this analysis was demonstrated with the B3 binding sites of the gene *AGL15*. Its transcription regulator, SHI2, interacts with a protein that contains the domain WD40. Transcription regulators SHI2, ARF1, and ARF5, which bind to the B3 sites in *AGL15*, were analyzed. 

A considerable portion of regulatory genetic variants are related to LOG analysis, domain features, DDIs, and motif binding preferences [39]. Integration of the ART FOUNDATION-LOG into DDI and domain–nucleotide interaction (DNI) analysis in GRNs will significantly enhance AI performance in assessing the impacts of genetic variants on phenotypic differences. This program is a versatile tool for the study of a wide spectrum of biological research subjects, which can be applied to other eukaryotic organisms. The program codes and datasets of the ART FOUNDATION-LOG are available for download at www.artfoundation.kr and https://sourceforge.net/projects/artfoundation-log/, accessed on 5 July 2023.

## 2. Methods

ART FOUNDATION-LOG consists of 7 DB modules that contain features extracted from existing databases. It also includes a simple program for analyzing regulatory factor–target gene interaction and a rough sketch for detecting nucleotide-containing ligand-binding motifs in proteins based on the NBDB [40].

### 2.1. Oligomerization Module

The ProtCAD provided protein assembly information derived from PDB entries. The PDB contains multiple plausible in vitro structures of protein assemblies that form homo- or hetero-oligomers by oligomerizing by themselves or with other proteins. It provides information on determinants of protein interactions, such as the stoichiometries and symmetries of clusters that belong to ProtCAD entries (GroupIDs) with the same Pfam architecture [23]. In this paper, we use the term “homo” to refer to clusters with the same sequence(s) and only one letter, “A”, in their stoichiometry, while “hetero” is used to refer to clusters with different sequences, e.g., “AB”, in their stoichiometry. To distinguish those without symmetry, we used C1_obligate_hetero_single_oligomer_obligate to refer to a cluster with only one type: C1 molecules from multiple sequences (e.g., C1-A2BC) and CMA for a cluster with C1-A1. The term “oligomer” is used to refer to a cluster with a number of 2 or higher, e.g., C2 or D3, in its symmetry. We created 19 feature variables based on 196 variables retrieved from ProtCAD (Table S1). 

### 2.2. DDI Module

3did provided sequences of interfaces from DDIs and DMIs [5]. We grouped entries with the same domain members into two clusters: one with non-redundant (NR) sets (e.g., Dom1Dom2) and the other with redundant sets (e.g., Dom1Dom2Dom1). We created a feature variable based on 13 values to measure the differences between the two clusters. These values mainly represented chemical properties such as the number of interacting domains, the number of interacting motifs (peptides), the number of amino acids in each fragment that indicate a sub-region of consecutive amino acids without gaps larger than 3, and the ProtCAD value (maximum of symmetries). They also contained information such as the means and standard deviations of the values in each cluster as well as the sizes of the memberships in each cluster. ANOVAs were performed regardless of the normality of the data because the magnitudes of the differences between two clusters were particularly important, but no non-parametric statistics with this capacity were available. 

The Plant-PrAS database provided genome-wide analyses of proteins in the context of the grand averages of hydrophobicity (GRAVYs), isoelectric points (pIs), binary presence/absence values of solubility, low complexity, protein secondary structural properties (b-sheets, IDRs, signal peptide(s), transmembrane helices, disulfide (S-S) bonds, and domain linkers), N/O-glycosylation sites, ubiquitination sites, functional regions (PASSs), peptide types (chloroplast transit, mitochondrial targeting, and secretory pathway signal peptides), and subcellular locations (E.R., chlo, mito, cysk, cyto, nucl, plas, extr, golg, pero, and vacu). Each domain feature variable derived from the Plant-PrAS database will be referred to as a “Plant-PrAS feature”. The qPTMplants database provided PTM information such as glycation, lysine, methylation, N-glycosylation, N-termini, O-GlcNAcylation, oxidation, persulfidation, phosphorylation, S-cyanylation, S-nitrosylation, and S-sulfenylation. Each domain feature derived from the qPTMplants database will be referred to as a “PTM feature”. We mapped these features to domains and interdomain regions based on protein domain positions provided by TAIR [27]. 

### 2.3. LLPS Module

DrLLPS includes approximately 40 distinct biomolecular condensates (Balbiani bodies, Cajal bodies, centrosome/spindle pole bodies, chromatin, chromatoid bodies, cleavage bodies, DDX1 bodies, DNA damage foci, droplets, Gemini of Cajal bodies, germ plasm/polar granules, histone locus bodies, insulator bodies, microtubules, mitochondrial RNA granules, neuronal granules, nuage, nuclear pore complexes, nuclear speckles, nuclear stress bodies, nucleoli, OPT domains, others, paraspeckles, P bodies, PcG bodies, pericentriolar matrices, perinucleolar compartments, P granules, PML nuclear bodies, post-synaptic density, pyrenoid matrices, receptor clusters, Sam68 nuclear bodies, siRNA bodies, spindle apparatuses, sponge bodies, stress granules, TAM bodies, and U bodies) [26]. LLPS-associated proteins are usually involved in the formation of multiple condensates. In the DrLLPS database, proteins are classified according to their associations with condensates, which resulted in 265 possible LLPS types (e.g., a protein specialized only in PML body formations, a protein involved in a number of LLPS nucleoli, nuclear speckles, Cajal bodies, centrosomes, etc.). In addition, DrLLPS provided three functional types of LLPS proteins: client, regulator, and scaffold. In this paper, they are referred to as LLPS factors. We created two different levels of variables: one with proteins as units and the other with domain dimers as units. At the protein level, we extracted entire domains belonging to proteins and counted the frequencies of the domains in the LLPS types. In the same way, we extracted functional types of protein and repeated the process. Considering that we had hetero-oligomers in different modules and that some large LLPS factors might have evolved from multiple genes, we created a variable to include partial matches to larger molecules in LLPS types and counted the numbers of Pfam assignments in the larger molecules. At the domain dimer level, we made lists of possible domain dimers and calculated their frequencies. We created nine feature variables altogether. In addition, we created feature variables, called “special flags”, based on 13 domain properties: the RNA binding domain; the DNA-binding domain (DBD); DMIs from 3did; and domains with low-complexity regions, disordered regions, repeats, coiled-coil structures, phosphorylation sites, and active sites, such as residues, that are responsible for catalysis. These special flags were created based on Pfam, D2P2, and DrLLPS (Table S1) [26,35,41]. 

### 2.4. GO Analysis Module

TAIR provided GO and PO data [28,29]. We created five categories: GO analyses, signaling pathways, gene associations, PO anatomy genes, and PO temporal genes. We retrieved 4 types of subcategories. The first one included attributes that involve signaling pathways: for example, hormones, response to light, and osmosensing. The second one included 34 major terms for GO analysis, such as cell communication and responses to abiotic stimuli, and the third one included words related to regulatory roles in annotation, e.g., enhancer, suppressor, chaperon, and activator. The last subcategory was original attributes of the database, e.g., acts_upstream_of_negative_effect and part_of. We created 10 feature variables that contained the frequency information of categories in the same way as those in the oligomerization module or the LLPS module (Table S1).

### 2.5. TF-Target Module

TF information for humans, *A. thaliana*, and peaches was provided by Cis-BP [30]. Cis-BP predicts the sequence preferences of TFs and measures correlations between DBD sequence similarities and DNA sequence preferences. We counted the number of types of TF-bound DNA motifs and how many TFs were DNA motif-bound. This information was incorporated in order to effectively search for TFs and TF targets with respect to their relationships to LOG.

### 2.6. PPI Module

The String and TcoF-DB databases provided PPI information for humans, *A. thaliana*, and peaches [31,42]. In addition, AtRegNet and Interactome 2.0 provided information for *A. thaliana* [27,43]. The LPInsider and NPInter databases provided interactions between proteins and RNAs [44,45]. The RNAs were grouped according to types such as lncRNA and miRNA. Domain assignment into transcripts was provided by GenBank, Gencode, TAIR, InterPro, and Pfam [27,35,36,46,47]. 

### 2.7. TF-to-Target Module

The TF binding sites in the targets were provided by the TF2DNA database for humans and by Yu et al. (2016) for *A. thaliana* [32,33]. The binding sites were mapped to gene features with the bedmap program [48]. The Gencode GFF and Ensembl GFF files were used for humans and *A. thaliana*, respectively [47,49]. Gene features included CDSs, exons, UTRs, introns, upstream and downstream regions, and the binding frequencies in each feature were counted. 

### 2.8. Proof of Concept of Search Algorithm

We created a rough sketch to study the interactions between protein domains and binding sites and between protein ligand-binding motifs and nucleotide-containing ligands. We also implemented a simple program to retrieve the regulatory elements in target genes and the domains (Pfams) of the protein pool in PPIs from the Plant Regulomics database. Pfam database provided hierarchical information of protein families; Pfams with evolutionary relationships were grouped to a set called Clan. To reduce the dimensions of the variables, amino acids were grouped according to the polarities and charges of their side chains (Table 1). Cysteine, glycine, histidine, and proline were considered to have special properties. According to properties of proteins of interest, group memberships may vary widely; e,g, each of the cysteine, glycine, histidine, and proline may make up a single membership group.

Using new amino acid group letters, the frequencies of the trimers in the DBDs were generated: PPP, PPN, PNP, …, RHR, and RRH.

For each DBD, Cis-BP provided ambiguous DNA sequences of the binding motifs in the target genes. DNA and ambiguous DNA were reassigned to DNA group letters (Table 2). Trimers of DNA group letters and their frequencies in binding motifs were generated.

The NBDB provided protein motifs (conserved sequence profiles) that interacted with 24 nucleotide-containing ligands (AMP, ADP, ATP, GMP, GDP, GTP, CTP, CoA, Acelyl-CoA, FMN, F-420, FAD(H), NAD(H), NADP, cyclic nucleotides and dinucleotides, cAMP, cGMP, c-di-AMP, c-di-GMP, and other biologically relevant cofactors (SAM, PPS, PAP, PLP, ThPP, and THD)) [40]. We converted NBDB member sequences, such as ENAGDTEAPT, into new amino acid group letters and created vector variables that contained the frequencies of the trimers of the new amino acid group letters per member sequence. Combinations of atoms and moieties belonging to 24 ligands were assigned into 11 groups: RBP, RBPF, RBPN, RBPS, RBPSO, RBSO, TOP, OP, TP, RPF, and RPFO (R: ribose; B: base; P: phosphate; F: flavin; N: nicotinamide; S: sulfur, and O: other moiety).

### 2.9. Demonstration of Program Usage

#### 2.9.1. Prediction of Oligomerization Types and LLPS Types

In the preparation of the datasets, we selected entries with multiple Pfam IDs from the intersection of three modules: oligomerization, DDI, and LLPS. We bisected the data based on the presence of Pfams associated with LLPS, which resulted in two datasets: LLPS and non-LLPS. The LLPS-type dataset contained the same contents as the LLPS dataset but had LLPS-type-related variables as a class. For each dataset, we used the same procedures as follows: We created feature variables based on arrays of related values retrieved from LOG modules (Table S1). Each feature variable based on an array of values was converted into a categorical variable by mapping or applying clustering algorithms such as EM, MakeDensityBasedClusterer, and SimpleKMeans in the WEKA program [50]. Most of the arrays were mapped to categorical variables without the application of clustering methods. Cluster memberships were values of the categorical variables. We removed redundancies from the dataset (non-redundant data). We used ten-fold cross-validation and percentage split (split 66% train, remainder test) methods. In addition, we used the stratified sampling method to split the non-redundant data into train and independent test sets and to save them in different files using the “StratifiedRemoveFolds” filter in Weka. In all classification runs, we performed the following procedures: Because some classes had a small number of instances, we applied either a “resample” or “SpreadSubsample” filter prior to classification. We applied the random forest classification algorithm with bagging, 100 iterations, base learner, and “print trees” option. The random forest architecture tree model was included in the prediction output. Details of parameter information are included in the prediction folders of the Supplementary Data. We performed hyperparameter tuning using the Weka experimenter interface. 

#### 2.9.2. Prediction of Oligomerization Types and Correlation Analysis

We selected TFs with multiple Pfam IDs from TF-target modules, retrieved the TF-interacting proteins from the PPI module, and selected only the proteins with LLPS properties, which will be referred to as LLPS factors. We retrieved information about the LLPS factors from LOG and TF-target modules (Supplementary Data: tf_llps_factor). The same procedures, that is, data exclusion, conversion to categorical variables, non-redundant data preparation, stratified sampling and train/independent test set creation, classification methods, and model evaluation, were applied to the tf_llps_factor dataset (Supplementary Data: Prediction_tf_llps_factor). In addition, a prediction-class distribution table of the probabilities of the oligomerization types each protein assembly formed was generated with the Weka program. We applied FAMD with the “FactoMineR” package in R [51]. The FAMD outputs contained information on the coordinates of data projected in principal dimensions: cos2, which was the quality of representation in principal dimension space, and contrib of variables, which was the contribution to the principal dimensions. We applied an association function in dython module from Python to calculate Pearson’s correlations. 

#### 2.9.3. Prediction of TF Binding Motif Types and PPI/PNI Study

We selected the target motif types of the TFs that interacted with the LLPS factors through physical contact in the second test and retrieved information from LOG modules and domain characterization information from DDI modules (Supplementary Data: co_tf_pras_ptm). The same preprocessing, classification, and evaluation methods were applied (Supplementary Data: Prediction_co_tf_pras_ptm). 

Integration of ART FOUNDATION-LOG into PPI/PNI analysis was demonstrated with the gene *AGL15*. We retrieved motif information and PPI data from Plant Regulomics with the query AT5G13790 [6]. For comparison, we also retrieved the data of the following genes: *ARF1* (AT1G23490), *ARF1* (AT1G59750), *ARF5* (AT1G19850), and *HSI2* (AT2G30470). Target gene motifs (B3 binding sites in promoter) and the binding factors (SHI2, ARF1, and ARF5 proteins) were compared regarding the following features: special flags, LLPS functional type, LLPS type, gene association category, GO analysis category, plantprASfeature, ptmfeature, PPI Clan pool, promoter motif class, and gene body motif class. All of these features were produced by the ART FOUNDATION-LOG core program, except for the last three features, which were generated by the retrieval program in the PNI proof of concept in the Methods section. 

## 3. Results

### 3.1. Prediction of Oligomerization Types and LLPS Types

Comparing the differences between the LLPS and the non-LLPS datasets, three oligomerization types, homo_hetero_moderate_oligomer_obligate, homo_obligate_monomer_oligomer_moderate, and homo_obligate_monomer_obligate, only belonged to proteins in the LLPS dataset and not to those in the non-LLPS dataset. The formation and dissolution of the LLPS were dynamic and correlated with the concentrations of proteins and nucleotides. Therefore, it seemed reasonable for higher occurrences of the proteins that formed both oligomers and monomers—those ending in “monomer_oligomer_moderate”—to be in the LLPS dataset. The proteins starting in “homo_hetero_moderate” had higher occurrences in the LLPS dataset. LLPS data were imbalanced; the percentages of the instances in five classes were 1.6%, 2.5%, 1.4%, 0.88%, and 0.080%. Therefore, the prediction accuracy of the model developed with the stratified sampling method was low—64%,while those with the cross-validation and the percentage split methods were 82% and 77%, respectively (Table S2, Supplementary Data: Prediction_llps). The prediction accuracies of non-LLPS models with the cross-validation, the percentage split, and the stratified independent test set were 86%, 68%, and 61%, respectively (Table S3, Figure S1, Supplementary Data: Prediction_non_llps).

The models tested with the cross-validation and the percentage split methods had accuracies of 83% and 81% in predicting the LLPS types, respectively (Table S4, Supplementary Data: Prediction_llps.type). Six classes had a small number of instances: 2.0%, 2.8%, 2.2%, 0.090%, 0.09%, and 0.03%. Therefore, the accuracy of the model that was trained with the stratified train set and tested with the stratified independent test set was low—64%. (Table S4, Supplementary Data: Prediction_llps.type). A tree model was included in each of the classification outputs (Supplementary Data: Prediction_llps, Prediction_non_llps, Prediction_llps.type). Model evaluation metrics of LLPS, LLPS-type, and non-LLPS models were included in Tables S2–S4, respectively.

### 3.2. Prediction and Extraction of Important Features from TF-LLPS Factor Data

The models tested with the cross-validation, the percentage split, and the stratified independent test set had accuracies of 83%, 80%, and 71% in predicting the oligomerization types, respectively (Table S5, Figure S2, Supplementary Data: Prediction_tf_llps_factor). The number of classes was four, where two classes had one and three instances. In addition, oligomerization-type distributions were calculated by AI algorithms in the Weka library; an example is given in Table S6. Caution should be taken in selecting the proteins to be included in a dataset. An estimation of the credibility of the accuracy of this method needs to be addressed. Correlation analyses showed that the domain dimer feature from ProtCAD, the features of the binding interfaces of the DDIs from 3did, the flags, the LLPS types, the LLPS functional types, and the numbers of the domains of the LLPS factors had relatively high correlations with the oligomerization types (corr > 0.6). We applied FAMD and plotted the coordinates of the variables in the first and the second principal dimensions; the variables of the TF-target motifs were located near the variables from the oligomerization and LLPS modules (Figure S3). 

### 3.3. Prediction of TF Binding Motif Types and PPI/PNI Study

Non-redundant data had twenty classes, of which fourteen classes had only one instance. After removing one member classes, the prediction accuracies of predicting the binding motif types of the TFs that the LLPS factors interacted with were calculated. The prediction accuracies with the cross-validation, the percentage split, and the stratified independent test set were 93%, 88%, and 91%, respectively (Table S7, Figure S4, Supplementary Data: Prediction_co_tf_pras_ptm). An association study with the Hotspot algorithm showed that the Plant-PrAS feature and the PTM feature of TFs had associations with binding motifs. The presence/absence values of multiple oligomerization types showed associations with binary variable–DMI flags. 

Plant Regulomics showed that ARF1 (AT1G23490), ARF1 (AT1G59750), ARF5 (AT1G19850), and HSI2 (AT2G30470) factors bound to the B3 binding sites in the promoter of *AGL15*. The Clan pool of the PPI of the binding factors, the SHI2, ARF1, and ARF5 proteins, showed domains with similar characteristics: ubiquitin, DNA-binding pseudoBarrel, and β-strand richness. All proteins except for AT1G59750 had Pkinase in the pool. While AT1G23490 and AT2G30470 were predicted to have LLPS properties (LLPS-related regulators and clients), AT1G59750 and AT1G19850 had non-LLPS clients. Most of them may have acted as transcription suppressors and/or activators. AT1G23490 and AT2G30470 were predicted to form various condensates in the centrosome, cytoplasm, and nucleus. In contrast, AT1G59750 and AT1G19850 were predicted to form only nucleoli. GO analysis showed that the binding factors responded to environmental stresses, light, hormones, and chemicals and were involved in signal transduction. They might have formed glycosylation, and contained β sheet and disordered regions. The peach homologs for *AGL15* (AT5G13790), *ARF1* (AT1G23490), *ARF1* (AT1G59750), *ARF5* (AT1G19850), and *HSI2* (AT2G30470) were *PAVAGL15* (Prupe.2G023100), NA, *ARF* (Prupe.1G585200), *ARF* (Prupe.1G368300), and Prupe.6G041000. *PAVAGL15* (Prupe.2G023100) played a key role in flower bud development. Both Prupe.1G585200 and Prupe.1G368300 regulated transcription via oligomerization. Prupe.1G585200 negatively regulated auxin response genes by forming oligomerization. The ART FOUNDATION-LOG may promote the identification of important regulatory elements and interaction partners of *PAVAGL15*, which may play crucial roles in structure development in peach.

## 4. Discussion

The Plant-PrAS features had associations with the properties of different functional types of LLPS. For example, LLPS factors that contained WD40 domains had a high chance of serving as scaffold types. Some of their partner TFs had domain linkers, S–S bonds, IDRs, β sheets, low-complexity regions, glycosylation, and ubiquitination, all of which, except for the domain linkers, belonged to the LLPS factors (scaffold) themselves. Interestingly, the TFs that interacted with the LLPS factors all had phosphorylation. The TFs that contained WD40 are involved in the transcription activation of anthocyanin-synthesis-related structural genes in barley [52]. A number of different LLPS scaffolds and regulators seemed to manage coordinated interactions for anthocyanin synthesis, transport, and storage, in which Natural Deep Eutectic Solvent (NADES) was speculated to be used as an inert solvent, suggesting highly complex regulatory processes [53]. As the physicochemical properties of liquid condensates remain largely unknown, in vitro experiments on them may encounter problems involving partial information. As the interaction of liquid condensates is a relatively new research topic, the standard methods in molecular biology and downstream analysis may require the implementation of new protocols and algorithms. Although current technology may have limitations in providing complete information, it may offer practical information for biomarker development. Structural properties that contribute to LLPS formation or satisfy the constraints imposed by LLPS, which pose impacts on DNA binding sites, may be roughly estimated with comparative studies using AI models based on a large number of factors that indicate cellular processes retrieved from numerous databases. The GO analysis showed that proteins that contain WD40 are part of the histone deacetylase complex, nuclear pores, vesicle coat, ubiquitin ligase complex, preribosomes, and spliceosomes and enable the following in *A. thaliana*: DNA-binding transcription factors, histone binding, kinase binding, protein heterodimerization and homodimerization activity, kinase activity, phosphatase regulators, ribosome binding, the signaling receptor complex, structural molecule activity, and transcription cis-regulatory regions (Table S8). WD40 may form important structural platforms for proteins that are involved in epigenetic activities. In the same test, the transcription repressor protein, SHI2, was known to interact with proteins that contain WD40 and DNA_binding-pseudoBarrel. Three ARF proteins also interacted with similar types of proteins with β-strand-rich domains and DNA_binding-pseudoBarrel [54,55,56,57]. This may provide important information for the elucidation of the modes of PPIs/PNIs. Integration of the ART FOUNDATION-LOG into GRN construction will promote the identification of phenotype-linked genetic variants. It is beyond the scope of this article to make inferences about PPIs/PNIs in peaches; this remains to be studied further. 

The Plant-PrAS features and PTM features supplemented the limited representational power of the flags. For instance, the homeobox, bZIP, and TCR TF families included TF members that interacted with LLPS factors and had domains with coiled-coil regions. These coiled-coil regions showed strong associations with DBDs and domains that contained an Intepro annotation called “activity”. The canonical coiled-coil regions had a heptad repeat structural motif [58]. The domains involved in oligomerization, such as the leucine zipper, the N-terminus of the homeobox, and the helix–loop–helix (HLH) proteins, also contained repeats. Although a considerable portion of TF families have various repeats in their oligomerization domains, the repeat flag is only equipped with the capacity to detect domains defined as repeats. The addition of flags with the detection capacity for domains that contain such repeats and half-sites will improve the program’s performance. Considering that the cellular in vivo environment is dynamic, with a large number of constantly changing factors, making predictions based on multiple variables from modules rather than a single variable, such as oligomerization type (e.g., homo_obligate_monomer_oligomer_moderate), will increase the accuracy of predicting interaction modes in PPI. The proofs of concept of the search algorithms in the Methods section may be implemented in PPI/PNI analysis with the additions of structural elements of nucleotides such as repeats and G-quadruplex and of protein domains; this remains to be carried out in further research [59,60,61,62,63].

## 5. Conclusions

Identifying genetic variants associated with phenotypes in GWAS data is challenging due to the complex nature of biological systems. AI applied to GRN with PPI/PNI interaction information will enable us to detect patterns underlying perplexing GWAS data. ART FOUNDATION-LOG will provide significant contribution to identification of important genetic variants.

## Figures and Tables

**Table 1:** Conversion table of amino acids.

Amino Acid Group Letter	Amino Acid	Amino Acid Features
P	R, K, S, T	Positive or polar uncharged
N	D, E, N, Q	Negative or polar uncharged
H	A, V, I, L, M	Hydrophobic
R	F, W, Y	Ring structures
S	C, G, P, H	Special properties

**Table 2:** Conversion table of DNA/ambiguous DNA.

DNA Group Letter	DNA/Ambiguous DNA
G	G
Z	R, S, K, B, D, V
X	A, C, T, Y, W, M, H
N	N

## Data Availability

The ART FOUNDATION-LOG was written in the Java programming language. The program codes, datasets, models, and outputs from the AI models are available for download at www.artfoundation.kr, accessed on 5 July 2023, and https://sourceforge.net/projects/artfoundation-log/, accessed on 5 July 2023.

## Supplementary Materials

The following supporting information can be downloaded at: https://www.mdpi.com/article/10.3390/genes14071476/s1. All datasets and program codes can be downloaded at www.artfoundation.kr, accessed on July 5 2023. Figure S1: Attribute matrix of the non-LLPS dataset; Figure S2: Attribute matrix of the TF-LLPS factor dataset; Figure S3: FAMD output: the projected coordinates of the variables from LOG, DDI, and TF-target modules in TF-LLPS factor data; Figure S4: Attribute matrix of the co_tf_pras_ptm dataset with the variable of Y-axis limited to TF motif family. A-L: feature variables from ProtCAD; M: cell loc; N: 3did; O-X: DrLLPS; Y, Z: Cis-BP; A1-H1: TFs from Plant-PrAS; I1-N1: TFs from qPTMplants; O1-Q1: LLPS factors from Plant-PrAS; S1, T1: LLPS factors from qPTMplants; U1-D3: LLPS factors/TFs from Cis-BP; and E3, F3: TFs from Cis-BP. Y-axis: TF motif family. Table S1: Variable and feature lists; Table S2: Model evaluation metrics: LLPS dataset; Table S3: Model evaluation metrics: non-LLPS dataset; Table S4: Model evaluation metrics: LLPS-Type dataset; Table S5: Model evaluation metrics: tf_llps_factor dataset; Table S6: Prediction class distribution table; Table S7: Model evaluation metrics: co_tf_pras_ptm dataset; Table S8. Lists of functions from GO analysis of proteins containing WD40.
